# The Etiology of Dental Caries

**Published:** 1898-01

**Authors:** D. M. Cathell

**Affiliations:** Chicago, Ill.


ARTICLE VI.

### THE ETIOLOGY OF DENTAL CARIES.

BY D. M. CATHELL, D. D. S., CHICAGO, ILL.

Read before the Chicago Dental Society, June, '96.

Caries of the teeth is known to have existed before what we call historic times. Caries of the teeth has been known and recognized ever since history began. It is the most prevalent of all diseases known attacking the human animal. In 1754 Jourdain and Bourdet, two scientific men of the times, regarded caries of the teeth as caused directly by "inflammation of the bone" or dentine. Later, John Hunter says it is the most common disease known to the teeth and calls it "mortification and something else."

In 1806 Dr. Fox supposed the cause to be an inflammation of the lining membrane of the pulp chamber, or what we know now as the odontoblastic layer. The inflamed area cut off the circulation in the dentine and gangrene resulted.

Mr. Bell in 1825 considered inflammation of the tooth substance itself was the cause of decay and beginning just under the enamel, then penetrating toward the pulp. The cause of the inflammation may have been a cold or other slight disturbance.

In 1830 Koecker conceived that the portion of dentine killed by inflammation was dissolved out by acids, the result of a putrefactive process.

In 1835 Robertson claimed that caries was caused by acids formed by decomposition occurring at the exact spot of the breaking down tooth structure; also that caries always began on the outside surface of the tooth.

Regnard corroborated the claim of Robertson, namely, "the destruction of the teeth by decomposition."

Desirabode claimed that if acids caused caries the whole tooth would go rather than in sections.

John Tomes, the father of the histology of the teeth, proved dentine to be without blood circulation, hence there could be no inflammation, but proposed the chemico-vital theory of decay; that the tooth must lose all life sensation before the decomposition of lime salts. Tomes also gave us "the transparent zone," or zone of resistance, supposing that the pulp in trying to head off the approaching decay threw up breastworks in front of the encroaching enemy to prevent further ingress.

Dr. Bridgeman proposed the electric theory of decay dissolving away lime salts by the formation and action of electricity.

Dr. Watts' theory (chemical, similar to Robertson) differed some, namely, that caries of the teeth was produced by *mineral* acids, developed at the immediate point where the action was produced, and did the work of dissolution while in their nascent state. The acids proposed were hydrochloric, producing yellow decay; nitric, the white variety, and sulphuric, the dark or black caries.

During the years the last four or five named gentlemen were working on the theory of the origin of dental caries, Magitot, of Paris, was working along lines hoping to find a better cause than any heretofore suggested. He was a faithful worker, and advanced many points to help out in the solution of the work in hand. Just what his final conclusions were, I am not able to state at present.

In the middle of the present century micro-organisms began to be seriously connected with caries of the teeth. After 1875, a closer study of micro-organisms began, and the different species or nations were studied with more distinctness. In the latter part of the 60's Leber and Rottenstein discovered that the "granular matter" of Tomes and others were germs in the dentinal tubuli of carious teeth, as well as in the cavity already produced. But they did not know how closely connected they were with the causes of caries. They also told of a zone of resisting calcific deposit appearing to be thrown up to prevent further ingress of the carious agents. Later, about 1880, Mills and Underwood showed by a new staining process that this supposed "zone of resistance" was also micro-organisms, probably of a different species. Dr. Koch discovered a process of staining different species of germs with different colors, and Mills and Underwood took advantage of his discovery. Mills and Underwood proposed that decalcification was the result of acids; that the acids were secreted by the germs and the germs themselves destroyed the organic matter, using it as food. They did not prove the proposition.

About 1881, Dr. Miller instituted a series of experiments and proved that ptyalin had nothing to do with producing an acid condition of the saliva, as had been supposed heretofore, but that micro-organisms produced the acid. Miller also proved the physiology, so to speak, of micro-organisms: (1) That they had a digestive apparatus, (produced a digestive solvent); (2) that they were nourished, (assimilated food;) (3) that they eliminated poisons, (produced acids); and (4) that they had the power of reproducing their own kind. In other words, he proved that micro organisms could take hold of certain materials, change the chemistry of them for their own uses and throw off again the changed material or chemical. Miller proved that the germs produced the special acid that dissolved out the lime salts of the teeth; that the micro-organisms fed upon or digested the animal or organic matrix. In other words, the function of the micro-organisms in the production of caries of the teeth was proven; also that the particular secretion dissolving the lime salts was *lactic acid.* Miller *suggested* that the process of enamel decay was similar to caries of the dentine; and such it has been proven to be by Black and Williams, the strepto-cocci media germs forming about themselves a gelatinous substance and becoming attached to the enamel, under which the lactic acid is secreted, dissolving out the cement substance from between the rods, which soon fall out, allowing ingress of micro-organisms to the dentine.

Last winter Williams of England read a paper before an association of dentists in New York city on the "Pathology of Enamel," exhibiting the most beautiful microscopic specimens ever seen. They were so thin, some of them, as to represent only the thickness of a single enamel rod. Many of his specimens showed that destruction of enamel under the influence of micro-organisms was going on long before the naked eye, with the help of a fine pointed explorer, could detect a fault upon the enamel surface. His experiments have proven much that heretofore had only been surmised. Our own Black has done considerable work in the same line and many suggestions and thoughts that have been presented by him from time to time, within the last few years, have now been proven beyond a doubt to be facts by the scientific and positive display of Williams. Indeed, the thoughts and ideas and researches of the two men have been practically the same.

Williams showed distinctly, not once, but many times, that lactic acid may dissolve out the cement substance between the enamel rods, and one or more enamel rods drop out, and through the puncture in the enamel a large area of dentine may be affected, and yet the eye or the sharp point that the dentist may have in the way of an explorer is not able to detect any faulty spot upon the surface of the enamel at that point. Williams' specimens under the microscope showed it very distinctly.

The other questions are soon answered. "Why is caries so much more active in some mouths than in others?" "Well, why is the atmosphere in some rooms not so healthy as in others? The moisture or the juices of the body as exhibited in the mouth, in some are more healthful than in others, that is, they are not so corrosive, not so active, and it is the *surroundings* of the tooth that influence its destruction, nothing else.

"What changes take place where caries ceases its activity in mouths heretofore predisposed?" If we *knew* what changes, we would apply the remedy and *cause* the change in all mouths immediately and stop further decay. *We do not know it*; we will not know it for some time to come.

"Are there recognizable signs by which we can know whether or not caries will cease with advancing age?" Most decidedly not. We cannot prognosticate in that direction yet. If there are signs, we do not know them. In some mouths we find almost perfect teeth, but little decay. Where we would suppose from the general appearance of the patient that we would find rapid decay, the teeth are perfect. We find individuals strong and healthy to all appearances, robust in every way, with their teeth softening and dissolving rapidly. In mouths where we find the thin saliva without odor, which we would call from a physical examination, pure and healthy, the teeth are being dissolved rapidly. Again, in persons whose saliva is ropy, and we would suppose that it would be very deleterious to tooth structure, we find well developed, strong teeth that are not carious. In fact, we know a few things, and a great many other things we do not know.—*Dental Review*.

